# Occurrence of Professional Burnout and Severity of Depressive Symptoms among Cardiac Nurses: A Cross-Sectional Study

**DOI:** 10.3390/ijerph182212038

**Published:** 2021-11-16

**Authors:** Anna Larysz, Anna Prokopowicz, Michał Zakliczyński, Izabella Uchmanowicz

**Affiliations:** 1 Clinic of Cardiac Transplantation and Mechanical Circulatory Support, Department of Heart Diseases, Wroclaw Medical University, 50-556 Wroclaw, Poland; annalarysz@wp.pl (A.L.); michal.zakliczynski@umw.edu.pl (M.Z.); 2 Department of Nursing and Obstetrics, Faculty of Health Sciences, Wroclaw Medical University, 51-618 Wroclaw, Poland; anna.prokopowicz@umw.edu.pl

**Keywords:** cardiac nurses, depression, occupational burnout, quality of care

## Abstract

Nurses with depression are not only likely to suffer themselves, but it may have an impact on their coworkers and potentially the quality of care they provide. This study aimed to determine the prevalence of depression and its association with burnout in cardiac nurses. A group of 400 cardiac nurses (361 women and 39 men) was enrolled. The standardized tools such as Maslach Burnout Inventory (MBI), Beck Depression Inventory (BDI), and Patient Health Questionaire-9 (PHQ-9) were used. A high level of professional burnout regarding emotional exhaustion was observed in 53.3% of nurses, high depersonalization in 52.5%, and low personal accomplishment in 72.8%. PHQ-9 and BDI were shown to correlate significantly and positively with all three MBI subscales (*p* < 0.05). High depressive symptoms and occupational burnout were correlated with depression (*p* < 0.05). In conclusion, nurses were found to have high levels of depression and professional burnout, which may have resulted in a negative impact on the quality of patient care. Identification of burnout in cardiac nurses is necessary to consider interventions to prevent stress and depression.

## 1. Introduction

As defined by Maslach and Jackson [1] professional burnout is attributed to the phenomenon of “psychological emotional exhaustion, depersonalization, and a diminished sense of personal accomplishment that occurred among various professionals who work with other people in challenging situations”. It is a delayed response to chronic stress [2]. Certain challenging situations apply to professions such as a doctor or nurse, where personal involvement in a relationship with a suffering person is necessary and desirable. The extent of occupational burnout among health professionals is usually reported at moderate to high levels, and it is widely believed that the risk of burnout in this department is higher than in the general working population [3].

The burnout process is phased. At first, there is emotional exhaustion in which the person feels overstrained, emotional emptiness, and a decrease of mental and physical energy. The exhaustion component represents the fundamental individual dimension of burnout. This is followed by a cynical treatment of the mentees, which leads to a greater distance from patients. Additionally, there is a sense of lack of competence, lack of self-esteem, and lack of professional satisfaction [4]. Researchers have developed a tool—Maslach Burnout Inventory (MBI) which examines the phases of burnout described above. The scale has strong psychometric properties and is the most frequently used research tool to measure the phenomenon of professional burnout. Some researchers believe that the burnout phases can occur in various orders [5].

Historically, in the last century, it was believed that the professional environment of the “aid and care” sector which is the health care, social workers, and the police, is primarily threatened by the process of professional burnout [1,6] Oswin [7] even described the phenomenon of “professional depression” affecting some nurses in long-term care. Firth et al. [8] documented that this phenomenon is similar to emotional exhaustion in long-term care. Moreover, these studies proved the relationship between burnout and the individual personality traits of the participants [9].

Nowadays, the relationship between burnout and the individual characteristics of an employee, who is unable to adapt to stressful working conditions or even incorporates to the profession their individual traits that are conducive to rapid burnout, is also sought [10]. Duan-Porter et al. [11] demonstrated that the exhaustion dimension of burnout was associated with the interpersonal variability of depressive symptoms. The above studies show that depression and burnout are two similar albeit distinct conditions. The stronger the severity of the burnout, the closer the symptoms are to clinical depression. The main difference is that the burnout phenomenon only affects the professional sphere whereas depression permeates other areas of life [12].

Nursing is a profession that is particularly prone to burnout due to the stressful nature of work and the aggravating closeness of the service provider–recipient relationship [1,13,14,15]. For this reason, the phenomenon of the link between burnout and depression among nursing staff is being investigated (discussed) in the international scientific arena.

There are a number of factors that contribute to burnout in nursing staff, including sociodemographic (age, marital status, number of children, gender), environmental (length of working day, nurse-patient ratio, experience) and psychological (anxiety, stress, verbal abuse) [16]. A number of work-related factors affecting the burnout syndrome among nurses have also been described, such as the deterioration of work team climate and effectiveness, and job security and satisfaction [17]. Moreover, Galanis et al. [18] in their systematic review and meta-analysis involving 18,935 nurses considered risk factors for burnout during the COVID-19 pandemic. They observed that the main risk factors for increased burnout were younger age, reduced social support, low family and colleague coping with the COVID-19 pandemic, increased feelings of insecurity, prolonged time working in quarantine areas, working in a high-risk environment, working in hospitals with inadequate material and human resources, increased workload, and lower levels of specialist training addressing the COVID-19 [18].

A positive correlation between stress, depression, anxiety, and professional burnout among Iranian nurses was found [19]. A significant level (38%) of depression was reported among 3474 nurses in China [20]. In Hong Kong, similar levels of depression were observed in nursing students even before they began working [21]. On this basis, it can be assumed that the level of depression is an individual trait and is brought into the profession as a negative resource that may affect the occurrence of professional burnout in the future. Golonka et al. [22] showed that depression is the primary cause of professional burnout in the dimension of emotional exhaustion. Masanotti et al. [23] found that the relationship between burnout and depression was due to the link between depression and a sense of coherence. In contrast, Bianchi et al. [24] did not show any distinction between depression and burnout. Later review papers describe the state of knowledge suggesting that the distinction between burnout and depression is conceptually fragile [25]. A longitudinal study by Toker and Biron [26] demonstrated that the increase in symptoms of depression over time from T1 to T2 predicted an increase in the level of professional burnout from T2 to T3 and vice versa. In this study, the severity of depression was measured with the Personal Health Questionnaire-9 (PHQ-9) in which most of the items relate to the perceived fatigue [27].

The above dispute over the distinctiveness or cohesion of the two phenomena is important from the point of view of scientific theory. However, the practical aspect of the research shows that symptoms of depression and professional burnout of hospital nurses can have a negative impact on patients’ safety [28,29]. Nurses’ poor mental health affects the quality of provided healthcare, and it should be borne in mind that the nurse’s recipient is usually a sick person who needs empathic support and understanding. For this reason, researching burnout and depression in a professional group of nurses is particularly essential, regardless of which process occurred first.

A cross-sectional project investigating the relationship between burnout and depression among Polish nurses will complement the statistics of this phenomenon in a global and local context. Therefore, the primary aim of the study was to demonstrate the two-way relationship between depression and burnout, i.e., the effect of depression on burnout and the effect of burnout on depression. In turn, the secondary aim was to compare the relationship between professional burnout and severity of depression measured with two separate measuring tools.

## 2. Materials and Methods

### 2.1. Participants and Design

For this cross-sectional study, a group of 464 cardiac nurses was recruited by convenience sampling from December 2019 to August 2020 in cardiology departments of four public hospitals in Wroclaw (Poland), of which one was the university clinical hospital. We decided to choose cardiac nurses because of the specificity of this nursing specialty and a significant load during everyday professional work including duties in the departments of General Cardiology and Cardiac Surgery and Interventional Cardiology and Cardiac Intensive Care. All group cardiac nurses who were caring for cardiovascular patients in the participating hospitals were invited to participate in this study. Nurses who were diagnosed with any prior mental disorders were excluded from the study. To improve the quality of reporting observational studies, the manuscript was organized in a manner compliant with the Strengthening the Reporting of Observational Studies in Epidemiology guidelines—STROBE [30].

### 2.2. Data Collection

The paper version questionnaires were distributed to the group of 464 nurses by their managers, who also were responsible of their collection. Participation was voluntary, and respondents were completely anonymous. A total of 84% of the surveys collected were complete (missing data was 16%), which means that complete data from 400 nurses were included in the analysis. Researchers Anna Larysz and Anna Prokopowicz were responsible for entering data into the database using Microsoft Office Excel.

### 2.3. Outcomes and Measures

Sociodemographic and other related background data were collected using a self-developed questionnaire. Sociodemographic data consisted of gender, age, marital status, education, work experience, number of jobs, form of employment, monthly working hours, workplace system, place of residence. For the purpose of the study the following instruments were used: Maslach Burnout Inventory (MBI), Beck Depression Inventory (BDI), and Patient Health Questionaire-9 (PHQ-9).

#### 2.3.1. Maslach Burnout Inventory (MBI)

The Polish version of MBI was used to measure nurses’ professional burnout. This tool allowed to assess the level of professional burnout in three aspects (subscales): emotional exhaustion, depersonalization, and lack of professional accomplishments. The results on each of these subscales are expressed on a 0–100-point scale, where a higher score indicates a greater level of professional burnout. In addition, the general index of professional burnout was also calculated as an average of all three subscales. The cut-off values of the MBI indicating low, medium, and high level of burnout for emotional exhaustion (EE) subscale are 0–16, 17–26, and >26, respectively; for depersonalization (DEP) subscale are 0–6, 7–12, and >12, respectively; and for professional accomplishment (PA) subscale are 0–31, 32–38, and >38, respectively. The MBI questionnaire as a measure of professional burnout among nurses worldwide is a useful tool for determining the effectiveness of burnout reduction measures generated in health policy planning [31,32]. The MBI was validated in Polish by Pasikowski [33] and achieved the following Cronbach’s alpha coefficients for the scales: EE of 0.85, DEP of 0.60, and PA of 0.76.

#### 2.3.2. Beck Depression Inventory (BDI)

The BDI is a 21-item, self-report rating inventory that measures characteristic attitudes and symptoms of depression. The BDI takes approximately 10 min to complete, although clients require a fifth–sixth grade reading level to adequately understand the questions [34]. The cut-off values of the BDI score are as follows: 0–13—no depression; 14–19—mild depression; 20–28—moderate depression; and 29–63—severe depression. Internal consistency for the BDI ranges from 0.73 to 0.92 with a mean of 0.86 [35]. Similar reliabilities have been found for the 13-item short form [34] The BDI demonstrates high internal consistency, with the Cronbach’s alpha of 0.86 and 0.81 for psychiatric and non-psychiatric populations respectively [35]. We used the Polish adaptation of BDI in this study which has been conducted by Parnowski and Jernajczyk [36].

#### 2.3.3. Patient Health Questionaire-9 (PHQ-9)

The PHQ-9 is a standardized self-assessment questionnaire used for screening of depressive symptoms in the general population. The PHQ-9 consists of nine questions or rather criteria for assessing depressive disorders in accordance with DSM-IV. It enables both the diagnosis of depression and the assessment of the severity of its individual symptoms [37] The PHQ-9 questionnaire is short and easy to score. The cut-off values of the PHQ-9 score are as follows: 0–4—no depression; 5–9—mild depression; 10–14—moderate depression; 15–19—moderately severe depression; and 20–27—severe depression. The Polish version of PHQ-9 was used in this study. It was translated and validated under by Tomaszewski et al. [38] who showed significant positive internal consistency for the Cronbach’s alpha coefficients of 0.70. The PHQ-9 is a questionnaire consisting of nine main questions and one supplementary question. The answer to each question is scored on a scale from 0 to 3, depending on the frequency of occurrence of a given symptom in the last 2 weeks (3 points indicate the most frequent occurrence of the symptom). The maximum number of points that can be obtained is 27, which indicates the greatest possible severity of depressive symptoms. A score below 5 points is a norm, from 5 to 9 points indicates mild depression, from 10 to 14 points moderate depression, from 15 to 19 points moderately severe depression, and from 20 to 27 points severe depression [38].

### 2.4. Ethical Considerations

The research project was approved by the Bioethics Committee of Wroclaw Medical University, Poland (permission no. KB–164/2019). All nurses provided their written informed consent. Voluntary participation and data confidentiality were emphasized. The study was carried out following the Declaration of Helsinki and Good Clinical Practice.

### 2.5. Data Analysis

The analysis was performed in program R, version 4.0.3 (R Foundation for Statistical Computing, Vienna, Austria) [39]. The arithmetic means (M), medians (Me), standard deviations (SD), range of variation (extreme values—min and max), lower quartile (Q1), and upper quartile (Q3) were calculated for quantitative variables. In turn, the number (*n*) and percentage of occurrences (%) of each value were calculated. Correlations between quantitative variables were analyzed using Spearman correlation coefficient. In the analysis, the significance level of 0.05 was assumed. Thus, all values for *p* < 0.05 were interpreted as demonstrating significant correlations.

## 3. Results

### 3.1. Participants’ Characteristics

A group of 400 individuals from nursing personnel took part in this study, of which 361 (90.3%) were women and 39 (28.3%) were men. Most nurses were between 41 and 50 years old (34%) and between 51 and 60 years of age (28.3%). The majority of nurses had a medical high school education (*n* = 121, 30.3%), over 20 years of professional experience (*n* = 218, 54.5%), one workplace (*n* = 199, 49.8%), monthly working hours between 101 and 180 h (*n* = 224, 56%), 12-h shift system (*n* = 310, 77.5%), people in a relationship (*n* = 278, 69.5%), and living in a city with over 100,000 residents (*n* = 240, 60%). Characteristics of the study participants is presented in Table 1.

### 3.2. Professional Burnout (MBI)

Results of the professional burnout levels (MBI) on the EE subscale indicate that 213 out of 400 respondents (53.3%) had a high level, 122 participants (30.5%) had a medium level, and 65 individuals (16.3%) had a low level of emotional exhaustion. Results of the professional burnout levels (MBI) on the DEP subscale show that 210 out of 400 participants (52.5%) had a high level, 159 respondents (39.8%) had a medium level, and 31 individuals (7.8%) had a low level of depersonalization. Furthermore, results of the professional burnout levels (MBI) on the PA subscale indicate that 291 out of 400 respondents (72.8%) had a low level, 97 individuals (24.3%) had a medium level, and 12 participants (3%) had a high level of professional accomplishment (Table 2).

### 3.3. Depressive Symptoms (PHQ-9 and BDI)

Results of the severity of depressive symptoms (PHQ-9) indicate that 163 out of 400 respondents (40.8%) had no symptoms of depression, 132 individuals (33%) had mild depression, 67 respondents (16.8%) had moderate depression, 26 participants (6.5%) had moderately severe depression, and 12 individuals (3%) had severe depression (Table 2).

Results of the severity of depressive symptoms (BDI) indicate that 318 out of 400 respondents (79.5%) had no symptoms of depression, 45 individuals (11.3%) had mild depression, 24 participants (6%) had moderate depression, and 13 respondents (3.3%) had severe depression (Table 2).

### 3.4. Correlations between PHQ-9 and MBI

PHQ-9 significantly (*p* < 0.05) and positively (r > 0) correlates with all three MBI subscales, and thus the greater the severity of depression, the bigger the occupational burnout (and vice versa: the bigger the burnout, the greater the severity of depression). The strongest correlation was found between the level of depression and emotional exhaustion (r = 0.495, *p* < 0.001), slightly weaker with depersonalization (r = 0.416, *p* < 0.001), and the weakest with professional accomplishment (r = 0.271, *p* < 0.001).

### 3.5. Correlations between BDI and MBI

BDI significantly (*p* < 0.05) and positively (r > 0) correlates with all three MBI subscales, and thus the greater the severity of depression, the bigger the occupational burnout (and vice versa: the bigger the burnout, the greater the severity of depression). The strongest correlation was found between the level of depression and emotional exhaustion (r = 0.452, *p* < 0.001), slightly weaker with depersonalization (r = 0.408, *p* < 0.001), and the weakest with professional accomplishment (r = 0.179, *p* < 0.001).

## 4. Discussion

The epidemiology of depression is the subject of numerous analyzes in Poland and worldwide. A study conducted in the United States in 2001–2003 by The National Comorbidity Survey Replication (NCS-R) on a representative sample of 9282 adults revealed the prevalence of mood disorders throughout life at 20.8%; major depression occurred at any point in life in 16.6% of the adult participants, dysthymia in 2.5%, and bipolar disorder in 3.9%. [40]. In turn, the European Study of the Epidemiology of Mental Disorders (ESEMeD) conducted by Alonso et al. [41] shows that 14% of the participants reported experiencing a mood disorder throughout their lifetime. The ESEMeD study conducted by Gabilondo et al. [42] in Spain on a representative sample of 5473 individuals indicated that major depressive disorders occurred at any point in life in 10.6% of respondents, and in the last year in 4% of participants.

The prevalence of mental disorders in Poland was the subject of the EZOP3 study conducted in 2008–2011 on a representative sample of individuals between 18 and 64 years old [40]. According to the results of the study, major depression (defined on the basis of the DSM-IV classification) occurred in 3% of the respondents, significantly more often in women—4%—than in men—1.9%. The prevalence of minor depression was very low with the overall rate being 0.4%. Bipolar disorder type I occurred at any point in life in 0.1% of respondents, equally frequent in women and men. Moreover, a similar percentage of both genders had bipolar disorder type II—0.1%. Dysthymia occurred at any point in life in 0.6% of participants, more frequently in women—0.9%—than in men—0.4%.

Another source of information on the epidemiology of depression is the Global Burden of Disease study conducted by the Institute for Health Metrics and Evaluation (IHME) [43]. According to IHME estimates, depressive disorders (major depression—F32, F33— and dysthymia—F34.1—according to ICD-10) were the second most common mental disorder in 2017, right after anxiety disorders. In the European Union, 20.7 million individuals (4.2% of the population) suffered from them in 2017, and 4.5 million people (1% of the population) were affected by bipolar disorder. Estimates for Poland from 2017 indicate that depressive disorders occurred in 1 million individuals (2.8% of the population), and bipolar disorders in 288,000 people (0.8% of the population).

In this study, in the group of cardiac nurses, major depression measured with the PHQ-9 scale was 3%, and with the BDI scale was 3.25% which is consistent with the research conducted by Kiejna [40] on the Polish population. This study demonstrated also that the depressive symptoms (33% mild depression, 16.8% moderate depression, and moderately severe depression 6.5% measured with PHQ-9 and mild depression 11.3%, moderate depression 6% measured with BDI) among cardiac nurses was common and these data are consistent with research by other scientists [44,45,46].

The aim of this study was to examine whether depression enhances professional burnout and whether professional burnout intensifies depressive symptoms. Professional burnout is considered to be an important problem, both individual and social, because the discomfort is felt not only by the burned-out employee but also by colleagues, service recipients, patients, mentees as well as family and friends. The process of professional burnout is a phenomenon typical for people whose occupation is to help others [47]. According to Maslach and Jackson [1] professional burnout is the body’s cumulative response to a chronic stressful situation which is associated with work performed by a given person or contacts with other people. There are three consecutive elements that make up the entire process of professional burnout. These are: emotional exhaustion, depersonalization, and a reduced sense of professional accomplishment [4]. The scope of responsibilities and the ability to make decisions play a key role in the development of professional burnout in nurses. This burnout amplifies with the intensification of the tasks assigned to the nurse (excess of duties), and when the control over situations decreases (no possibility of making decisions) [1]. Professional burnout is a state of emotional, mental, and physical exhaustion caused by prolonged involvement in emotionally charged situations—it is associated with slow progression of mental deterioration [48].

So far, there is no research conducted among cardiac nurses, thus it is difficult to refer to the studies of other authors in this area. However, as much as 53.3% of participants had a high level of emotional exhaustion, 52.5% had a high level of depersonalization, and even more, because as much as 72.8% had a low level of professional accomplishment measured with MBI. Both the PHQ-9 and BDI scores correlated significantly (*p* < 0.05) and positively (r > 0) with all MBI subscales, thus the more severe the depression, the greater the burnout, and vice versa. This means that working in cardiovascular settings is very demanding and affects a high level of professional burnout.

For instance, Piko [49] stated that in Hungary, nurses have the highest level of burnout in the emotional domain, and similarly, in the research conducted by Uchmanowicz et al. [50], emotional exhaustion was the main determinant of professional burnout. This is consistent with the results of the presented study, where emotional exhaustion was strongly correlated with the severity of depressive symptoms in both assessments with PHQ-9 (r = 0.495, *p* < 0.001) and BDI (r = 0.452, *p* < 0.001) tools. Piko [49] showed that emotional exhaustion was strongly correlated with job dissatisfaction (*p* < 0.001). Also, job satisfaction was a negative predictor of each type of burnout subscale (*p* < 0.001), role conflict was an organizational factor positively affecting emotional exhaustion (*p* < 0.001) and depersonalization (*p* < 0.001). In turn, Uchmanowicz et al. [50] showed a significant positive correlation (*p* < 0.05) between the level rationing of nursing care (BERNCA-R) and burnout syndrome (MBI) and a significant negative correlation (*p* < 0.05) between the level rationing of nursing care (BERNCA-R) and the job satisfaction (JSS). Additionally, the emotional exhaustion domain was found to be an independent predictor of the rationing of nursing care (*p* < 0.05). Based on the above-mentioned studies, the results of our study should be extended to include the importance of organizational factors and predictors affecting the incidence of care rationing among cardiac nurses.

Such results may suggest that it is necessary to consider interventions which may aim to improve working conditions and therefore prevent stress and occupational burnout in order to reduce and prevent the symptoms of depression.

Taking into account the factors contributing to professional burnout, primary prevention should be implemented in every workplace in order to avoid the occurrence of this phenomenon. It should be based on estimating the risk of mental and physical stress and eliminating those components that generate stress as much as possible. It is also about all the changes in the work environment that lead to a reduction in tensions. Secondary prevention, on the other hand, refers to the individual—the employee. It is based on acquiring the ability to cope with difficult and stressful situations. The last type of prevention is tertiary prevention, which involves the treatment of stress-related health effects [51].

### 4.1. Study Limitations

The limitations of this research include the small study group (*n* = 400) as well as the selection of nurses from only one region of Poland (Lower Silesian Voivodeship). Hence the caution in assigning these results to the profile of all cardiac nurses nationwide and worldwide. Our study did not address the negative implications of burnout on quality of care; therefore, the self-esteemed quality of nursing care provided and its relationship with the results of the surveys conducted should be considered in future studies. Moreover, the results of our study should be extexnded to include the importance of organizational factors and predictors affecting the incidence of care rationing among cardiac nurses. However, despite these limitations, we consider such studies important because of the lack of research conducted on nurses working in cardiovascular settings.

### 4.2. Practical Implications

The results of the study indicate that the prevalence of cardiac nurses with depression and professional burnout is high and it is still a serious problem for health care, which should be identified urgently in order to prevent a decrease in the quality of patient care. Identification of professional burnout in cardiac nurses is necessary to consider interventions to improve working conditions and therefore prevent stress and burnout in order to reduce and prevent the symptoms of depression. This knowledge will enable considering the factors contributing to professional burnout, primary prevention should be implemented in every workplace in order to avoid the occurrence of this phenomenon. It is also necessary to strive for greater attention to the reported problems of nurses, both professional and personal nature and to strengthen the care of cardiac nurses, in search of experiences in the production of well-being at work and appropriate atmosphere.

## 5. Conclusions

The nurses have high levels of depression and professional burnout, which may have resulted in a negative impact on the quality of patient care. It is very important to recognize this problem because patient care is heavily reliant upon their ability to deliver the best care for possible.

## Figures and Tables

**Table 1:** Sociodemographic and work-related characteristics of the nurses (*n* = 400).

Characteristic		Total (*n* = 400)
Age (years)	20–30	69 (17.3%)
	31–40	82 (20.5%)
	41–50	136 (34.0%)
	51–60	113 (28.3%)
Gender	Female	361 (90.3%)
	Male	39 (9.8%)
Education	Medical high school	121 (30.3%)
	Medical study	55 (13.8%)
	Bachelor degree	116 (29.0%)
	Master degree	107 (26.8%)
	Other	1 (0.3%)
Work experience (years)	0–5	66 (16.5%)
	6–10	40 (10.0%)
	11–15	44 (11.0%)
	16–20	32 (8.0%)
	>20	218 (54.5%)
Number of works	1	199 (49.8%)
	2	141 (35.3%)
	3	35 (8.8%)
	4	13 (3.3%)
	>5	12 (3.0%)
Work hours per month	<100	6 (1.5%)
	101–180	224 (56.0%)
	181–230	114 (28.5%)
	231–300	39 (9.8%)
	>300	17 (4.3%)
Work system	8-h shift work	72 (18.0%)
	12-h shift on duty	310 (77.5%)
	Other	18 (4.5%)
Marital status	Alone	92 (19.0%)
	Formal relationship	278 (69.5%)
	Informal relationships	30 (7.5%)
	Widow/widower	16 (4.0%)
Place of residence	City > 100 000 residents	240 (60.0%)
	City < 100 000 residents	100 (25.0%)
	Village	60 (15.0%)

Abbreviations: *n*, number of participants.

**Table 2:** Results of standardized tools: MBI, PHQ-9, and BDI.

Research Tool	*n*	Missing Data	M	SD	Me	Min	Max	Q1	Q3
MBI: EE	400	0	27.37	9.3	28	0	52	22	34
MBI: DEP	400	0	13.48	5.07	14	0	28	10	16
MBI: PA	400	0	26.97	6.85	28	0	44	24	32
PHQ-9	400	0	6.91	5.38	6	0	27	3	10
BDI	400	0	7.95	8.3	5	0	44	1	12

Abbreviations: MBI, Maslach Burnout Inventory; EE, emotional exhaustion; DEP, depersonalization; PA, personal accomplishments; BDI, Beck Depression Inventory; PHQ-9, Patient Health Questionaire-9; *n*, number of participants; M, mean; SD, standard deviation; Me, median, Min, minimum, Max, maximum; Q1, quartile 1st; Q3, quartile 3rd.

## Data Availability

The data presented in this study are available on request from the corresponding author (I.U.).
